# Identification and Validation of Hub Genes Associated with Bladder Cancer by Integrated Bioinformatics and Experimental Assays

**DOI:** 10.3389/fonc.2021.782981

**Published:** 2021-12-20

**Authors:** Kang Chen, Ji Xing, Weimin Yu, Yuqi Xia, Yunlong Zhang, Fan Cheng, Ting Rao

**Affiliations:** Department of Urology, Renmin Hospital of Wuhan University, Wuhan, China

**Keywords:** bladder cancer, differential gene expression analysis, weighted gene co-expression network analysis, protein–protein interaction network, survival analysis, tumor microenvironment, TCGA, GEO

## Abstract

Bladder cancer (BC) is the most common malignant tumor of the urinary system and is associated with high morbidity and mortality; however, the molecular mechanism underlying its occurrence is not clear. In this study, the gene expression profile and related clinical information of GSE13507 were downloaded from the Gene Expression Omnibus (GEO) database. RNA sequencing (RNA-seq) expression data and related clinical information were retrieved from The Cancer Genome Atlas (TCGA) database. Overlapping genes were identified by differential gene expression analysis and weighted gene co-expression network analysis (WGCNA). Then, we carried out functional enrichment analysis to understand the potential biological functions of these co-expressed genes. Finally, we performed a protein–protein interaction (PPI) analysis combined with survival analysis. Using the CytoHubba plug-in of Cytoscape, *TROAP*, *CENPF*, *PRC1*, *AURKB*, *CCNB2*, *CDC20*, *TTK*, *CEP55*, *ASPM*, and *CDCA8* were identified as candidate central genes. According to the survival analysis, the high expression of *TTK* was related to the poor overall survival (OS) of patients with BC. *TTK* may also affect the bladder tumor microenvironment (TME) by affecting the number of immune cells. The expression level of *TTK* was verified by immunohistochemistry (IHC) and real-time quantitative polymerase chain reaction (RT-qPCR), and the tumor-promoting effect of *TTK* in BC cells was confirmed *in vitro*. Our results also identified the MSC-AS1/hsa-miR-664b-3p/TTK regulatory axis, which may also play an important role in the progression of BC, but further research is needed to verify this result. In summary, our results provide a new idea for accurate early diagnosis, clinical treatment, and prognosis of BC

## Introduction

Bladder cancer (BC) is a highly malignant tumor that is associated with high morbidity and mortality (1). Most BCs are urothelial cancers; among patients with these cancers, approximately 75% lack muscular invasion and 25% have muscle invasion or metastasis (2). In particular, myometrial invasive BC has poor prognosis and often relapses after the first resection (3). Therefore, it is of great significance to fully evaluate and identify prognostic factors to better understand BC. BC lacks clinically useful biomarkers to predict the stage and clinical outcome of the disease (4, 5). Therefore, there is an urgent need to explore the molecular pathogenesis of BC and identify biomarkers closely related to its diagnosis, occurrence, progression, and prognosis.

In recent years, bioinformatics has become increasingly popular in gene expression profile analysis and molecular mechanism research. Weighted gene co-expression network analysis (WGCNA) is an advanced method for constructing co-expression modules based on similar gene expression patterns and analyzing the relationship between modules and specific features (6). In addition, another powerful analysis in transcriptomics is differential gene expression analysis, which provides a method to study the molecular mechanisms of genomic regulation and to detect quantitative changes in the expression levels between the experimental group and the control group (7). This difference in gene expression may lead to the discovery of the underlying potential biomarkers for specific diseases. Therefore, this paper combines gene differential expression analysis and WGCNA to improve the recognition ability of highly related genes that can be used as candidate biomarkers.

The tumor microenvironment (TME) is the internal environment in which tumor cells are generated and grown, and it provides conditions for tumor occurrence, proliferation, invasion, and metastasis and is closely related to the survival of tumor cells (8, 9). Many studies have shown that the TME plays an important role in tumorigenesis and development and significantly affects the treatment response and clinical outcomes of cancer patients; tumor-infiltrating immune cells (TICs) in the TME are a promising index of the effects of treatment (10).

In this study, WGCNA and differential gene expression analysis were carried out on the messenger RNA (mRNA) expression data of BC in The Cancer Genome Atlas (TCGA) and Gene Expression Omnibus (GEO) databases to identify differentially co-expressed genes. We further explored the mechanisms related to the development of BC through functional enrichment and protein–protein interaction (PPI) analyses combined with survival analysis. Finally, after a series of screening and verification tests, we identified a threonine and tyrosine kinase (*TTK*) gene that can indeed promote tumor progression and predict the prognosis of BC, and we revealed that *TTK* may be a potential indicator of the changes in the TME in BC.

## Materials and Methods

### Acquisition of Raw Data

**Figure 1:**
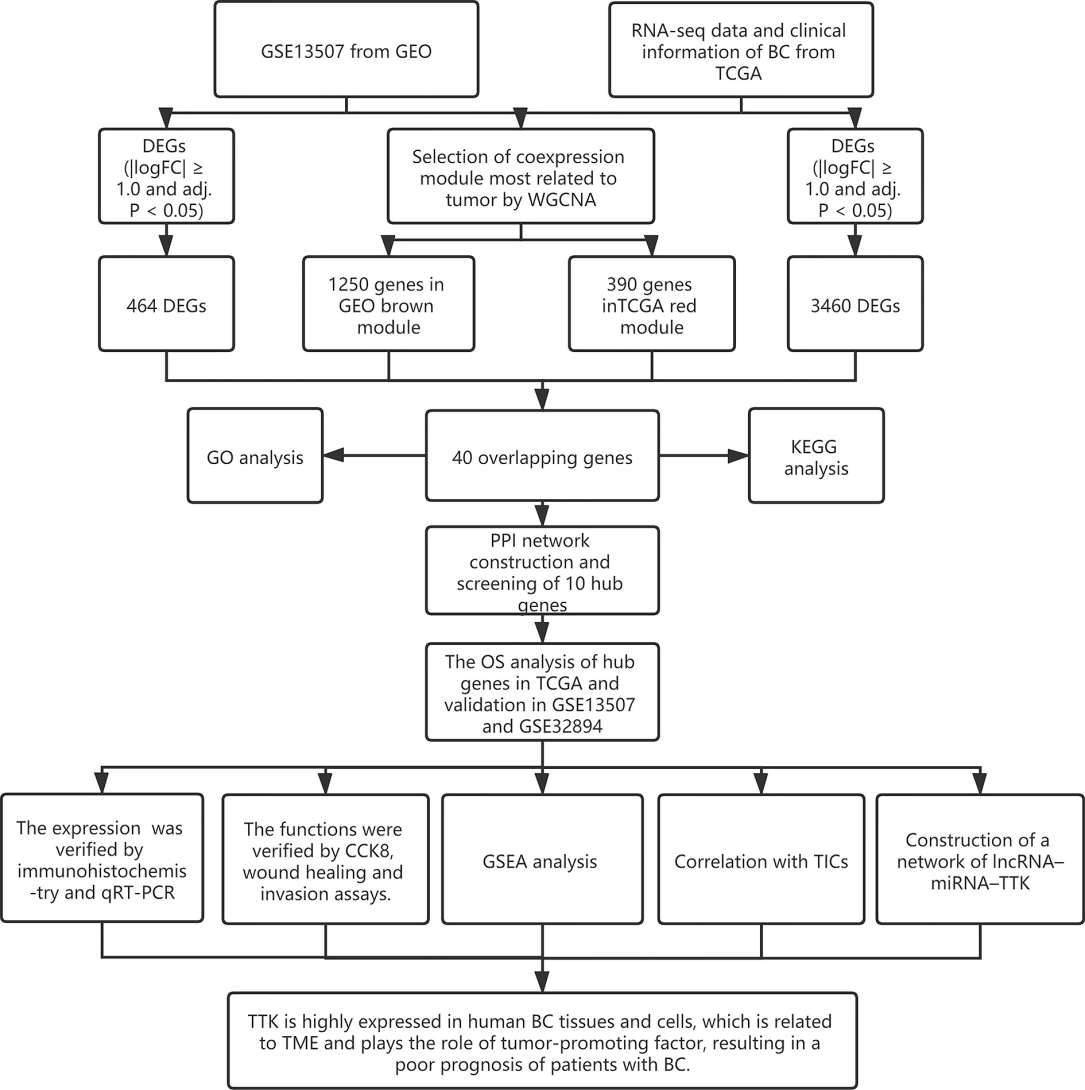
Research flowchart.

The research process is shown in **Figure 1**. The gene expression profile of BC was downloaded from TCGA (https://portal.gdc.cancer.gov/) and GEO (http://www.ncbi.nlm.nih.gov/geo/). In TCGA database, all BC data and the corresponding clinical information were downloaded free of charge through the R package TCGAbiolinks (11). A total of 433 bladder tissue samples were collected, including 414 BC samples and 19 normal samples. After excluding 6 duplicate samples and 2 samples without complete survival time and status, 406 of 414 tumor samples had complete clinical information and were included for further study. In this study, we retained genes with CPM (counts per million) ≥1. After filtering using the RPKM function (gene count divided by gene length) in the Edger package, a total of 14,829 genes with RPKM values were analyzed in the next step. In addition, we used the R packet GEOquery (12) to obtain another normalized expression profile of bladder gene expression (GSE13507) from the GEO. GSE13507 consists of 188 tumor samples and 68 normal tissues from patients with BC. According to the annotation file, the probes are converted into gene symbols, and the repeated probes of the same gene are removed by determining the expression median of all the corresponding probes. As a result, 24,343 genes were selected for follow-up analysis. In addition, we used 224 samples with survival information from the GSE32894 308 tumor samples as the prognostic validation cohort of hub genes. The clinical characteristics of the three datasets are shown in **Supplementary Table S1**.

### Identifying a Co-expression Module by WGCNA

WGCNA is a method for analyzing multi-sample gene expression patterns. WGCNA can cluster genes with similar expression patterns and analyze the relationship between modules and specific traits or phenotypes. Based on the gene expression data map of TCGA-BC and GSE13507, the co-expression network was established using the WGCNA R package (6). Pearson’s correlation matrix was constructed with the gene correlation coefficient, and the weighted adjacency matrix was transformed into a weighted adjacency matrix using the power function *a_ij_* = |*S_ij_* |*^β^* (adjacency relationship between *a_ij_* = gene *i* and gene *j*, Pearson’s correlation between *S_ij_* = gene *i* and gene *j*, and *β* = soft threshold). To satisfy the scale-free distribution, the soft thresholds were set to *β* = 3 and 11 and transformed into a topological overlap matrix (TOM) and the corresponding degree of dissimilarity (1-TOM). Then, the hierarchical clustering treemap of the 1-TOM matrix was constructed, and genes with similar expressions were classified into different gene co-expression modules. WGCNA included 14,513 genes in the TCGA-BC dataset and 8,889 genes in the GSE13507 dataset, with a total of 17,058 genes.

### Differential Expression Analysis and Interaction With Modules of Interest

To identify the differentially expressed genes (DEGs) between BC tissues and normal tissues, LIMMA (13) was used to screen DEGs in the TCGA-BC and GSE13507 datasets. Genes with the cutoff criteria of |logFC| ≥ 1.0 and *p* < 0.05 were considered as DEGs. The R packet ggplot2 was used to visualize the DEGs of the TCGA-BC and GSE13507 datasets as a volcano map. Then, the overlapping genes and co-expressed genes between the DEGs extracted from the co-expression network were used to identify potential prognostic genes. The R-packet VennDiagram (14) was used to represent them as Venn maps.

### Function Enrichment Analysis

The clusterProfiler package (15) is rich in functions for Gene Ontology (GO) and Kyoto Encyclopedia of Genes and Genomes (KEGG) (16). GO annotation contains three sub-ontologies—biological processes (BPs), cellular components (CCs), and molecular functions (MFs)—that identify the biological properties of the genes and genomes of all organisms (17). An adjusted *p*-value <0.05 was considered to be statistically significant.

### PPI Network and Hub Gene Identification

A search tool (STRING, https://string-db.org/) for searching a database of interacting genes was used to construct a PPI network (18). According to a confidence level ≥0.4, genes with significant interactions were identified, and any genes not related to other genes were excluded. The screening results were input into Cytoscape software 3.8.2 (19) for network visualization. In the co-expression network, the maximum clique centrality (MCC) algorithm is considered to be the most effective method to identify the central node (20). The MCC of each node was calculated through the CytoHubba plug-in in Cytoscape. In this study, the 10 genes with the highest MCC values were considered to be hub genes.

### Verification of the Prognostic Value of Hub Genes

To verify the prognostic value of hub genes, we used the survival package in R software to conduct Kaplan–Meier univariate survival analysis based on the data in TCGA to explore the relationship between overall survival (OS) and hub gene expression. In our study, only patients who completed the follow-up time were selected for survival analysis, and these patients were divided into two groups according to the median expressions of the hub genes. The survival-related hub genes with logarithmic rank *p* < 0.05 were considered to be statistically significant.

### Verification of the Expressions of Hub Genes With Prognostic Value and Gene Set Enrichment Analysis

We further verified the expressions of hub genes with prognostic value between BC tissues and normal tissues using the GEPIA online website (http://gepia.cancer-pku.cn/) (21). The Hallmark and C7 gene set V6.2 set from the Molecular Signatures database (MSigDB) was downloaded as the target set for gene set enrichment analysis (GSEA) (22), and the GSEA-3.0 software downloaded from the Broad Institute was used for GSEA. Only the gene sets with false discovery rate (FDR) *q* < 0.05 were considered to be meaningful when the GSEA was performed with all transcriptional groups of all the tumor samples in TCGA-BC.

### Tumor-Infiltrating Immune Cells

The CIBERSORT (23) computational method was applied to estimate the TIC abundance profile in all tumor samples, followed by quality filtering; only 201 tumor samples with *p* < 0.05 were selected for subsequent analysis.

### Competing Endogenous RNA Network Construction

The ENCORI (Starbase v3.0) database was used to predict the potential upstream microRNAs (miRNAs) and long non-coding RNAs (lncRNAs) that interact with *TTK*. The final competing endogenous RNA (ceRNA) network was further processed with Cytoscape software v3.8.2.

### Cell Culture

The human BC cell lines 5637 and T24 and the immortalized human bladder epithelial cell line SV-HUC-1 were derived from the Cell Bank of Shanghai, Chinese Academy of Sciences, and were cultured in Roswell Park Memorial Institute (RPMI) 1640 medium containing 10% fetal bovine serum (Scientific Cells, San Diego, CA, USA) and 100 U/ml penicillin–streptomycin (Gibco, Grand Island, NY, USA).

### Immunohistochemistry

We collected 20 samples from patients undergoing radical cystectomy in the Renmin Hospital of Wuhan University, and these samples included 10 human muscle-invasive urothelial carcinoma tissues and 10 adjacent normal bladder tissues. The samples were independently histopathologically confirmed by two pathologists. Each patient signed the informed consent form, and the medical ethics committee of our hospital approved the use of tumor tissue in this study. The collection of all the tissue samples was approved by the patients. After paraffin sectioning of the tumor tissues, the sections were incubated with an anti-TTK antibody (10381-1-AP, 1:100; Proteintech, Wuhan, China) at 4°C overnight. Then, paraffin sections were incubated with horseradish peroxidase (HRP)-coupled secondary antibodies at room temperature for 30 min. Subsequently, they were stained with DAB, and the tissues were counterstained with hematoxylin. Finally, photos were taken with an inverted microscope. The immunohistochemistry (IHC) intensity scores were as follows: 0 (negative), 1 (weak), 2 (medium), and 3 (strong). The cell positivity scores were 0 [≤ 1 (10%–25%)], 2 (26%–50%), 3 (51%–75%), and 4 (greater than 75%). The final score was determined by multiplying the strength score by the positive rate score. An IHC method and an imaging scheme were carried out, and the image was evaluated as described earlier (11).

### siRNA Transfection

The si-TTK used in this experiment was designed and synthesized by Shenggong Bioengineering (Shanghai) Co., Ltd. The 5637 and T24 cells were inoculated into a 6-well plate for culture, and when the cell density reached 50%, the cells were transfected according to the instructions in the Lipofectamine 2000 transfection kit. The cells were divided into a *TTK* knockdown group (si-TTK) and a negative transfection group (si-NC). Lipofectamine 2000 was mixed with si-TTK or si-NC (final concentration, 50 nM) and then added to the cells. After incubation for 6 h, the media were replaced with a complete culture media for 24 h. The small interfering RNA (siRNA) sequences were: 5′-CGGGAACUGUUAACCAAAUUATT-3′ (sense) and 5′-UAAUUUGGUUAACAGUUCCCGTT-3′ (antisense). A negative control (NC) was transfected simultaneously with the siRNA. The NC sequences were: 5′-UUCUCCGAACGUGUCACGUTT-3′ (sense) and 5′-ACGUGACACGUUCGGAGAATT-3′ (antisense).

### RNA Extraction and Real-Time Quantitative Polymerase Chain Reaction

To evaluate the expression levels of *TTK*, miR-664b-3p, and MSC-AS1, we extracted total RNA from 5637 cells, T24 cells, and SV-HUC-1 cells using the RNA TRIzol reagent (Invitrogen, Carlsbad, CA, USA). Reverse transcription was carried out using the iScript cDNA Synthesis Kit from Bio-Rad. Real-time quantitative PCR (RT-qPCR) was conducted on a LightCycler^®^ 480 Real-Time PCR System (Roche, Mannheim, Germany). The relative gene expression levels were calculated using the 2^−∆∆Ct^ method with *GAPDH* or *U6* as an endogenous control. The primer sequences for each gene are shown in **Supplementary Table S2**.

### Cell Counting Kit-8 Assay

The 5637 and T24 cells transfected with si-NC and si-TTK were inoculated in 96-well plates at a density of 2,000/well, with triplicate wells in each group, and the day the cells were seeded was recorded as 0 day after cell attachment. Then, the optical density (OD) values were measured after incubation with the Cell Counting Kit-8 (CCK-8) reagent (medium: CCK-8 = 10:1) for 2 h, and this measurement was conducted at 1, 2, 3, and 4 days. The experiment was repeated three times.

### Wound Healing Assay

The 5637 and T24 cells transfected with si-NC and si-TTK were seeded in a 6-well plate. When the cell density reached 90%, scratches were made vertically on the bottom of the well with a 200-µl sterilized pipette tip. After washing with phosphate-buffered saline (PBS), a complete cell culture medium was added and the cells cultured in an incubator. At 0 and 48 h, 6 visual fields were randomly selected, cell images were collected using an inverted phase-contrast microscope, and the same point of the wound was selected for comparison at different times. Wound healing was detected and the ability of cells to migrate was measured.

### Transwell Assays

In the cell invasion experiment, 20,000 si-NC- and si-TTK-transfected 5637 and T24 cells were collected and transferred to the upper chamber of a Transwell plate. The RPMI-1640 medium (200 µl) was added to the upper chamber and 600 µl complete medium added to the lower chamber. The noninvasive cells on the upper part of each compartment were removed after 48 h. The Transwell chamber was then fixed in 4% phosphate-buffered neutral formalin for 30 min and stained with 1% crystal violet.

### Statistical Analysis

SPSS version 18.0 (Chicago, IL, USA) and GraphPad Prism version 7.0 software (La Hora, CA, USA) were used for statistical analyses. Quantitative data are presented as the average ± SD. The double-tailed Student’s *t*-test was used to evaluate the differences between two groups. A *p* < 0.05 indicates a statistically significant difference.

## Results

### Construction of a Weighted Gene Co-expression Module

**Figure 2:**
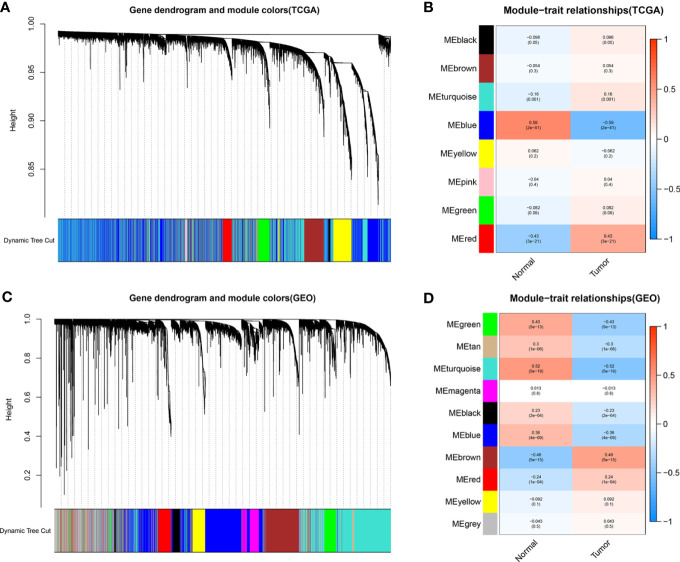
Identification of modules related to clinical information in The Cancer Genome Atlas breast cancer (TCGA-BC) and GSE13507 datasets. **(A, B)** According to the topological overlap (1-TOM) **(A)** of the TCGA-BC datasets and the correlation heatmap between module features and traits including normal and tumor **(B)**, the tree diagrams of the co-expression network modules are clustered in different degrees. **(C, D)** According to the topological overlap (1-TOM) **(C)** of the GSE13507 dataset and the correlation heatmap between module features and traits including normal and tumor **(D)**, the tree diagrams of the co-expression network modules are clustered in different degrees.

To identify functional clusters in BC patients, gene co-expression networks were constructed from the TCGA-BC and GSE13507 datasets using WGCNA packages. Each module was assigned a color, and a total of 8 modules in TCGA-BC (**Figure 2A**) and 10 modules in GSE13507 (**Figure 2C**) were identified in this study. Then, we generated a heatmap of the module–feature relationship to evaluate the association between each module and two clinical features (cancer and normal). The results of the module–feature relationship are shown in **Figures 2B, D**, which revealed that the red module in TCGA-BC and the brown module in GSE13507 had the highest correlations with BC organization (TCGA red module: *r* = 0.43, *p* = 3e−21; GEO brown module: *r* = 0.46, *p* = 5e−15).

### Gene Intersection Between Differentially Expressed Genes and Co-expression Modules

**Figure 3:**
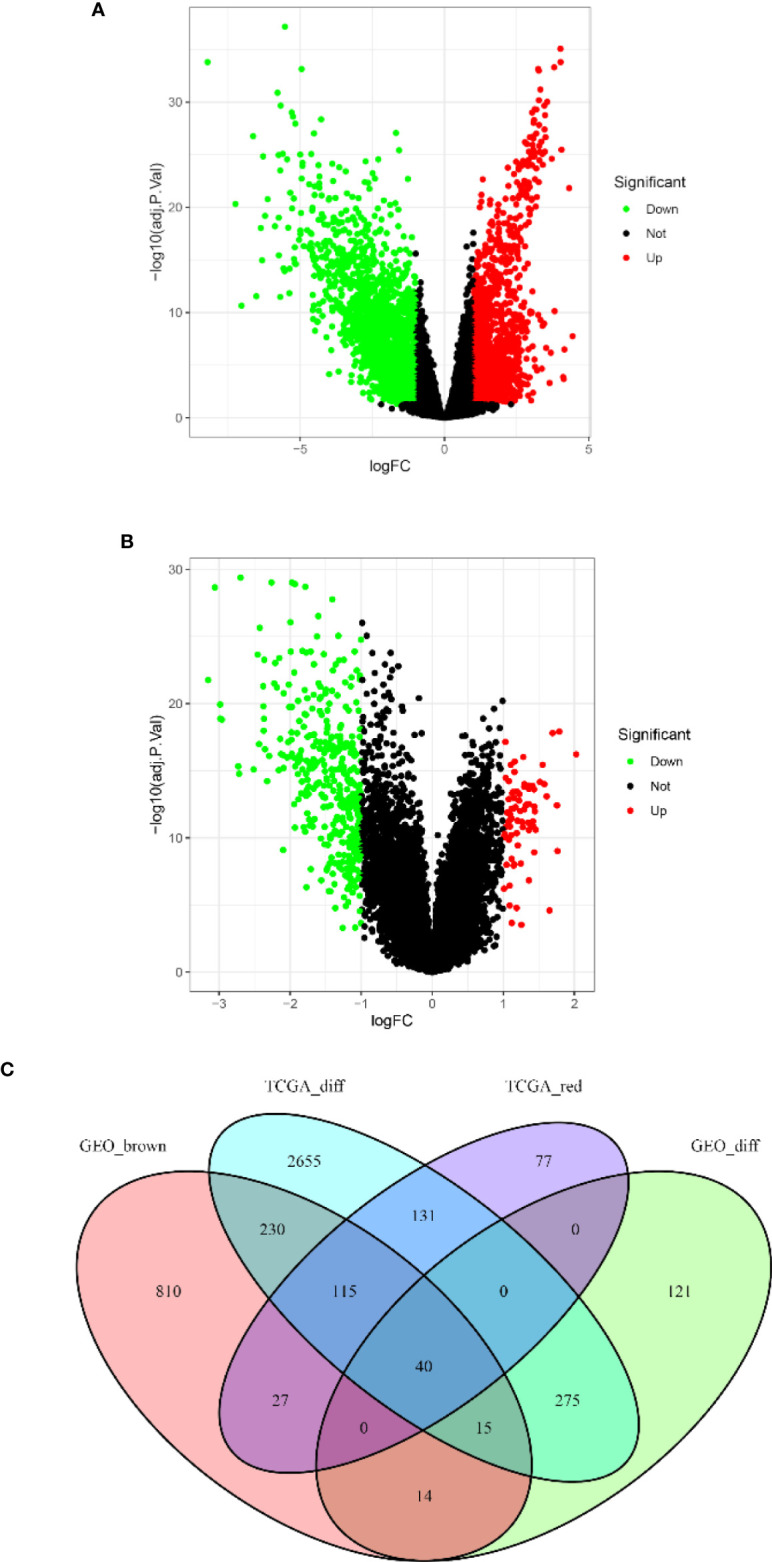
Volcano map of the differentially expressed genes (DEGs) of breast cancer (BC) patients and the gene Venn map of the list of DEGs. **(A)** Volcano map of DEGs in The Cancer Genome Atlas breast cancer (TCGA-BC) dataset. **(B)** Volcano map of DEGs in the GSE13507 dataset. Upregulated and downregulated genes are represented by *red* and *green dots*, respectively. **(C)** There are 40 overlapping genes at the intersection of the list of DEG and the two co-expression modules.

Based on the cutoff criteria of |logFC| ≥ 1.0 and adj.*p* < 0.05, the LIMMA package showed that 3,460 and 464 DEGs were differentially expressed in the TCGA (**Figure 3A**) and GSE13507 datasets (**Figure 3B**), respectively. As shown in **Figure 3C**, 390 and 1,250 co-expressed genes were found in the red module of the TCGA dataset and the brown module of GSE13507, respectively. A total of 40 overlapping genes were extracted and used to verify the genes of the co-expression module (**Figure 3C** and **Supplementary Table S3**).

### Functional Enrichment Analysis of the 40 Overlapping Genes

**Figure 4:**
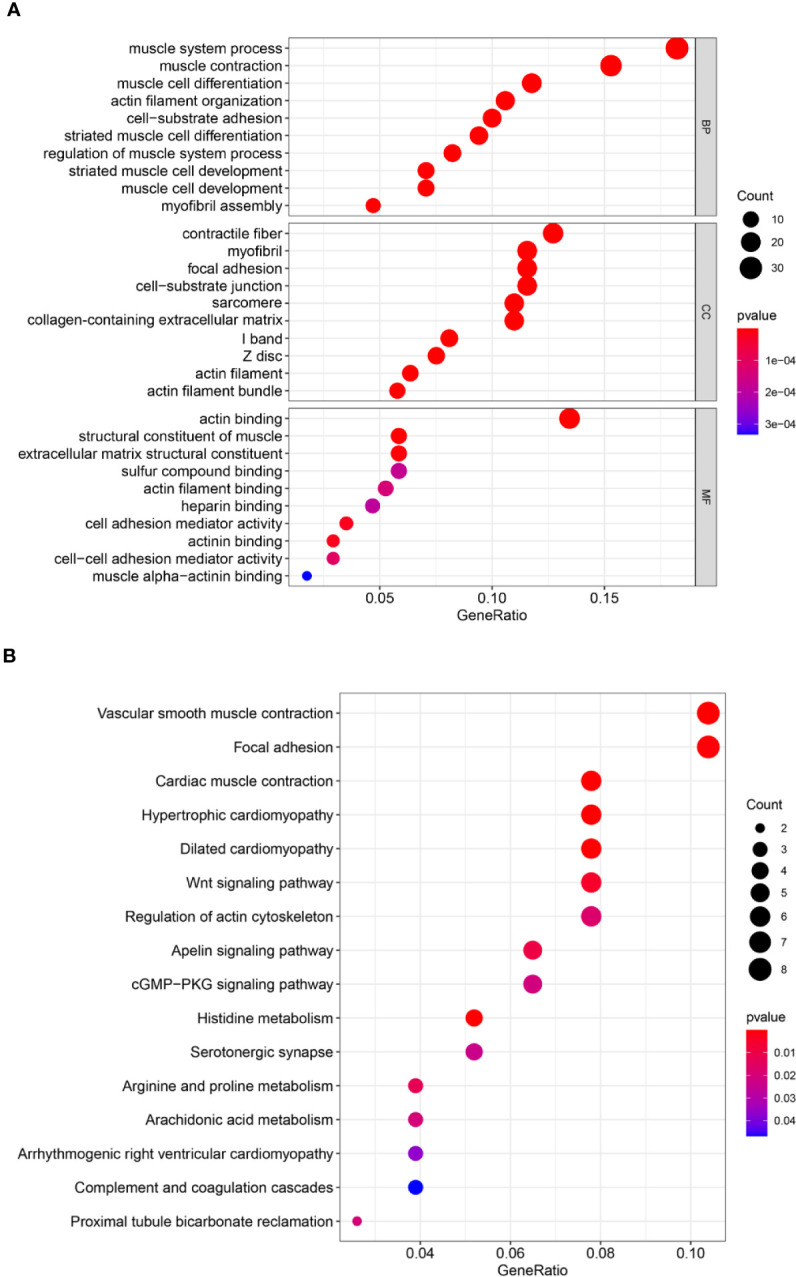
Forty genes were analyzed by Gene Ontology (GO) and Kyoto Encyclopedia of Genes and Genomes (KEGG) pathway analyses. **(A)** GO analysis. **(B)** KEGG pathway analysis. GO analysis includes biological processes (BPs), cellular components (CCs), and molecular functions (MFs). The *count* represents the number of genes and the *color* represents the adjusted *p*-value.

To identify the potential biological function and pathway correlation of these 40 genes, we analyzed the GO and KEGG pathways. GO enrichment analysis showed that these genes were significantly enriched in the muscle system process, contractile fiber, and actin binding (**Figure 4A**). KEGG analysis showed that these genes were enriched mainly in vascular smooth muscle contraction, focal adhesion, and cardiac muscle contraction (**Figure 4B**).

### Construction of PPI Network and Hub Gene Recognition

**Figure 5:**
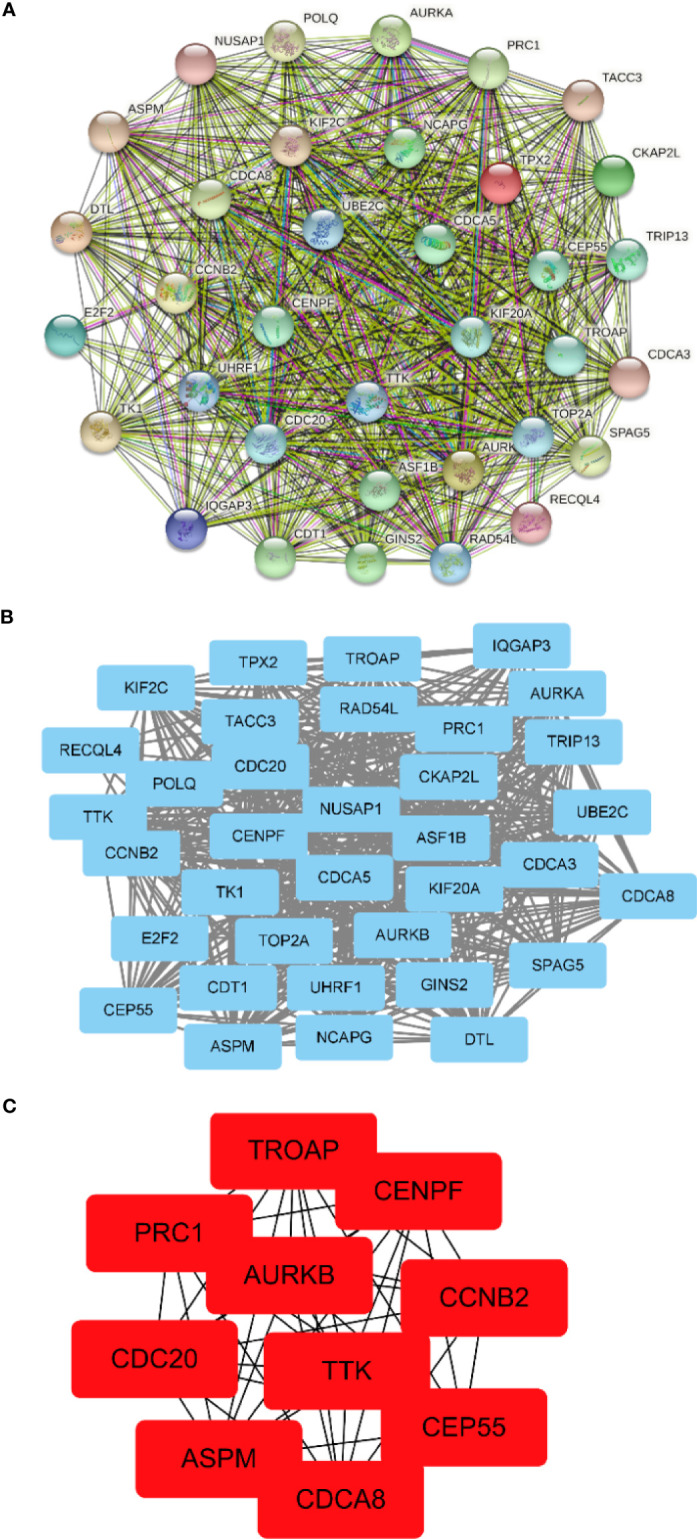
Construction of the protein–protein interaction (PPI) network and screening of hub genes. **(A)** PPI network constructed by the STRING database. **(B)** The PPI network is visualized by Cytoscape software. The *blue nodes* represent the genes. *Edges* indicate interaction associations between nodes. **(C)** Identification of the hub genes from the PPI network by the maximum clique centrality (MCC) algorithm. The *red nodes* represent genes with the highest MCC sores.

The PPI network between overlapping genes was established using the STRING database, in which 5 genes were excluded from the PPI network without any association with other genes (**Figure 5A**). **Figure 5B** shows visualization by importing the PPI network into Cytoscape software. According to the MCC algorithm of the CytoHubba plug-in, the 10 genes with the highest scores included *TROAP*, *CENPF*, *PRC1*, *AURKB*, *CCNB2*, *CDC20*, *TTK*, *CEP55*, *ASPM*, and *CDCA8* (**Figure 5C**).

### Verification of the Prognostic Value of 10 Hub Genes

**Figure 6:**
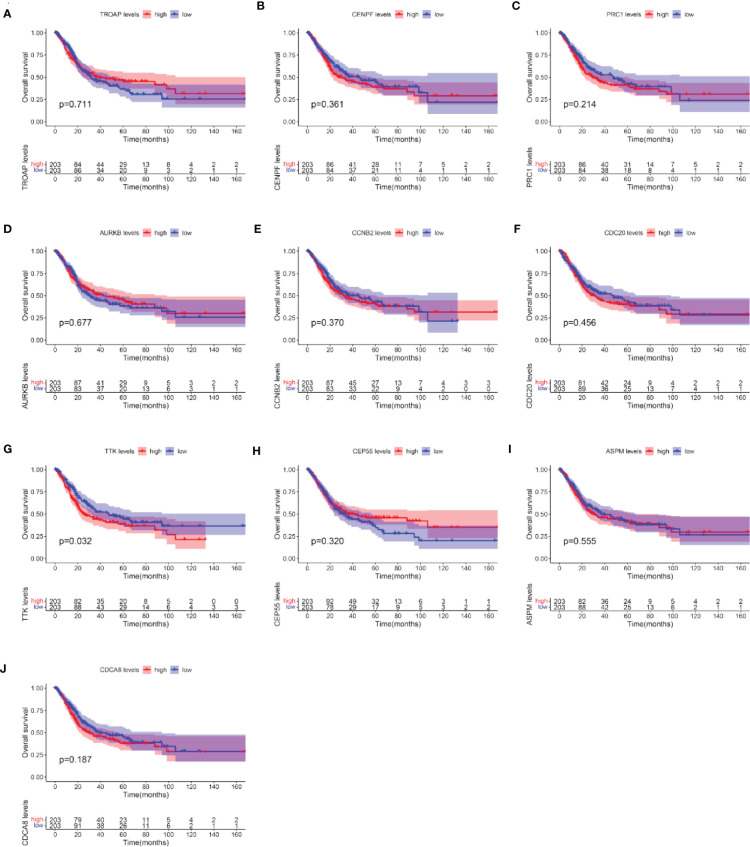
Overall survival (OS) analyses of the 10 hub genes. (A–J) *TROAP* **(A)**, *CENPF* **(B)**, *PRC1*(C), *AURKB* **(D)**, *CCNB2* **(E)**, *CDC20* **(F)**, *TTK* **(G)**, *CEP55* **(H)**, *ASPM* **(I)**, and *CDCA8* **(J)**. The *red line* represents samples with high hub gene expression; the *blue line* represents samples with low hub gene expression.

After the identification of 10 hub genes (*TROAP*, *CENPF*, *PRC1*, *AURKB*, *CCNB2*, *CDC20*, *TTK*, *CEP55*, *ASPM*, and *CDCA8*) using the CytoHubba plug-in, we verified the prognostic value of these 10 hub genes with survival-related data in TCGA. The OS analysis of the 10 hub genes was performed with the Kaplan–Meier plotter using the R survival package. The analysis showed that, among the 10 hub genes, only the expression level of *TTK* was significantly correlated with OS in BC patients (*p* < 0.05; **Figures 6A–J**). Our results showed that the high expression of *TTK* indicates worse prognosis of patients with BC.

### Validation of *TTK* and GSEA

**Figure 7:**
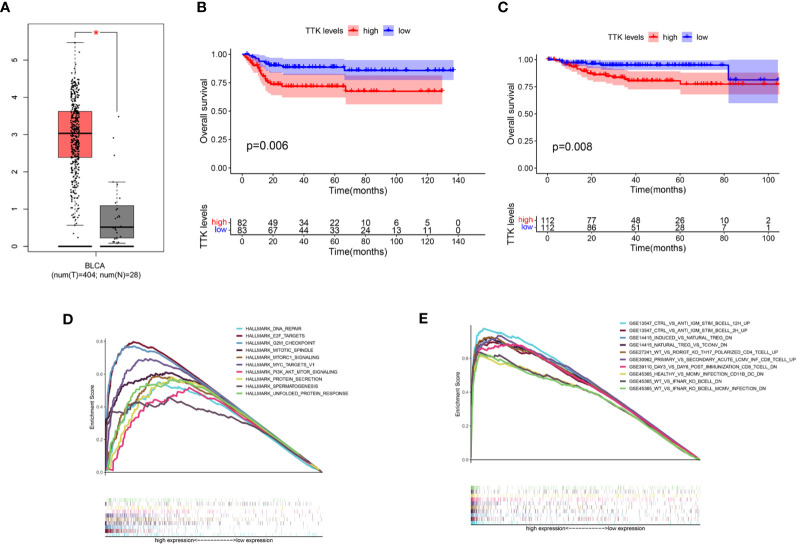
Differential expression, prognostic value, and gene set enrichment analysis (GSEA) of *TTK*. **(A)** Expression level of *TTK* in breast cancer (BC) and paracancerous normal tissues verified in GEPIA (*T*, tumor; *N*, normal). **(B, C)** Prognostic value of *TTK* verified with the GSE13507 **(B)** and GSE32894 **(C)** datasets. **(D)** A rich set of genes obtained by the Hallmark collection from samples with high *TTK* expression. Each *line* represents a specific set of genes with a *unique color*. Only the genes with false discovery rate (FDR) *q* < 0.05 are considered to be meaningful. **(E)** Through the samples with high *TTK* expressions, the immune gene set was enriched in the C7 collection. Only a few dominant gene sets are shown in the picture. *P < 0.05.

In light of the negative correlation between the expression level of *TTK* and the survival period of patients with BC, we verified the high expression and prognostic value of *TTK* in BC through GEPIA, GSE13507, and GES32894 (*p* < 0.05; **Figures 7A–C**) and used the median expression of *TTK* in the BC samples of TCGA as the cutoff point; samples with high and low expressions of *TTK* were analyzed using GSEA. As shown in **Figure 7D**, the results of GSEA using the Hallmark pathway database showed that the genes in the high-*TTK*-expression group were enriched mainly in DNA repair, E2F targets, G2M checkpoint, mitotic spindle, mtorc1 signaling, MYC targets v1, PI3K/AKT/mTOR signaling, protein secretion, spermatogenesis, and unfolded protein response. For the C7 set defined by MSigDB, the immune gene set and multiple immune function gene sets were enriched in the group with a high expression of *TTK* (**Figure 7E**). However, in the low-*TTK*-expression group, no gene set was enriched in either Hallmark or C7 enrichment analysis. The results suggest that *TTK* may be a carcinogenic factor and a potential indicator of the status of the TME.

### Correlation Between *TTK* and TICs

**Figure 8:**
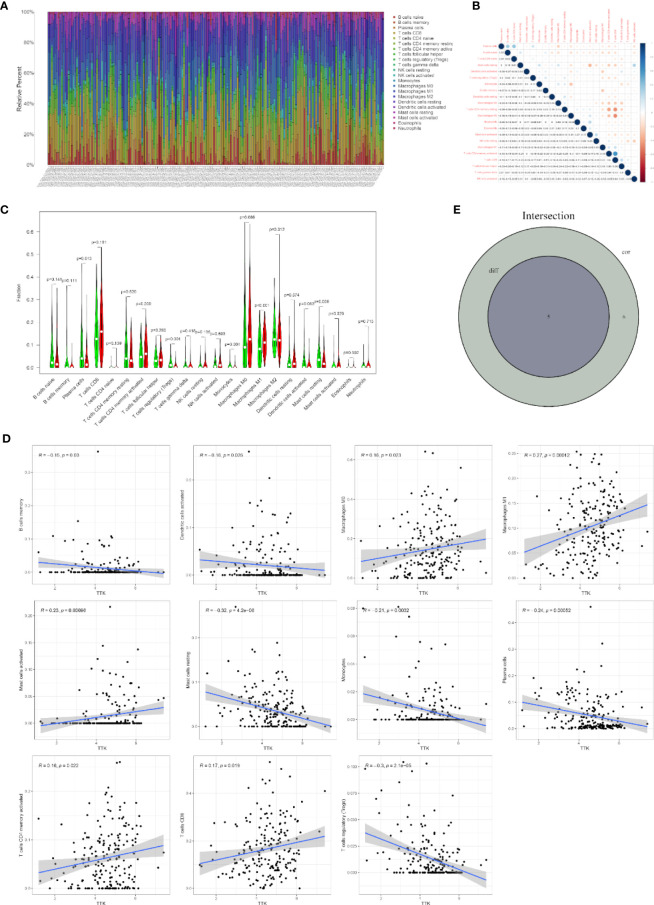
Correlation of infiltrating immune cell numbers with *TTK* expression. **(A)** Bar chart showing the proportions of 22 types of infiltrating immune cells in The Cancer Genome Atlas breast cancer (TCGA-BC) samples. The *column of the graph* is labeled with the sample ID of TCGA. **(B)** Heatmap of the correlation of 22 types of infiltrating immune cells in TCGA-BC samples. The *darker the color* or the *higher the value*, the stronger the correlation. **(C)** Violin plot showing the differences in the numbers of the 22 types of infiltrating immune cells in BC samples with low or high expressions of *TTK*. *Red* represents a high-expression sample and *green* represents a low-expression sample. **(D)** Scatter plots showing the correlation of the numbers of 11 types of infiltrating immune cells with *TTK* expression. **(E)** Venn diagram displaying the correlation of the numbers of five types of infiltrating immune cells with *TTK* expression co-determined by the difference and correlation analyses.

To further confirm the correlation between *TTK* expression and the immune microenvironment, the proportion of tumor-infiltrating immune subsets was analyzed using the CIBERSORT algorithm, and 22 types of immune cell profiles in TCGA-BC samples were constructed (**Figures 8A, B**). The results of the difference and correlation analyses (**Figures 8C, D**) showed that *TTK* expression was inversely proportional to the number of plasma cells, regulatory T cells (Tregs), and resting mast cells and directly proportional to the number of M1 macrophages and activated mast cells (**Figure 8E**). These results further support the effect of the levels of *TTK* on the immune activity of the TME.

### Identification of the Expression and Function of *TTK*

**Figure 9:**
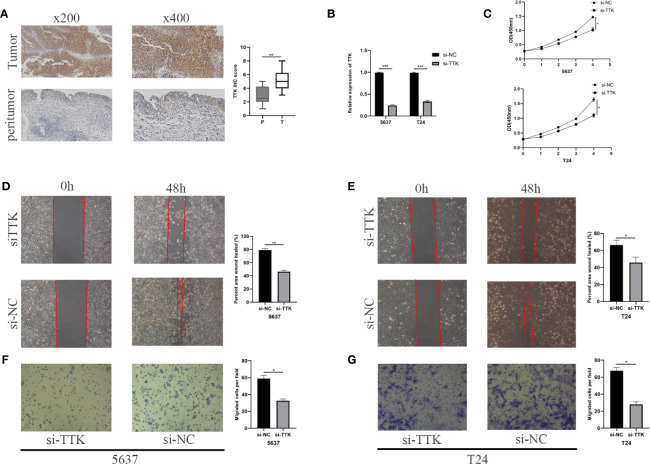
Analysis of *TTK*. **(A)** Representative *TTK* immunohistochemistry (IHC) staining and scores for P and T samples. *P*, peritumor; *T*, tumor. **(B)** RT-qPCR validation of *TTK* knockdown. **(C)** CCK-8 assay analysis of the proliferation of 5637 and T24 cells transfected with si-TTK (*TTK* knockdown) or si-NC (negative transfection). **(D, E)** Wound healing experiment evaluating the effect of *TTK* knockdown on the migration ability of 5637 and T24 cells. **(F, G)** Effect of *TTK* knockdown on the invasive ability of 5637 and T24 cells detected by the Transwell assay. *p < 0.05; **p < 0.01; ***p < 0.001.

We conducted an IHC assay to compare the expression levels of *TTK* between 10 BC tissues and 10 paracancerous tissues. As shown in **Figure 9A**, the expression levels of *TTK* were higher in BC tissues than those in paracancerous tissues. To study the function of *TTK in vitro*, we verified the expression levels of *TTK* in si-TTK-transfected 5637 cells and T24 cells by qPCR (**Figure 9B**). Using the CCK-8 assay, we analyzed the effect of *TTK* on cell proliferation. Our results showed that the downregulation of *TTK* expression significantly inhibited the proliferation of the two cell lines (**Figure 9C**). Subsequently, we also evaluated the effect of *TTK* on the migration and invasion of BC cells *in vitro*. The results showed that the downregulation of *TTK* expression significantly inhibited the migration and invasion of 5637 and T24 cells (**Figures 9D–G**). These results suggest that *TTK* plays an important role in the proliferation, migration, and invasion of BC cells.

### Construction of a Network of lncRNA–miRNA–*TTK*

**Figure 10:**
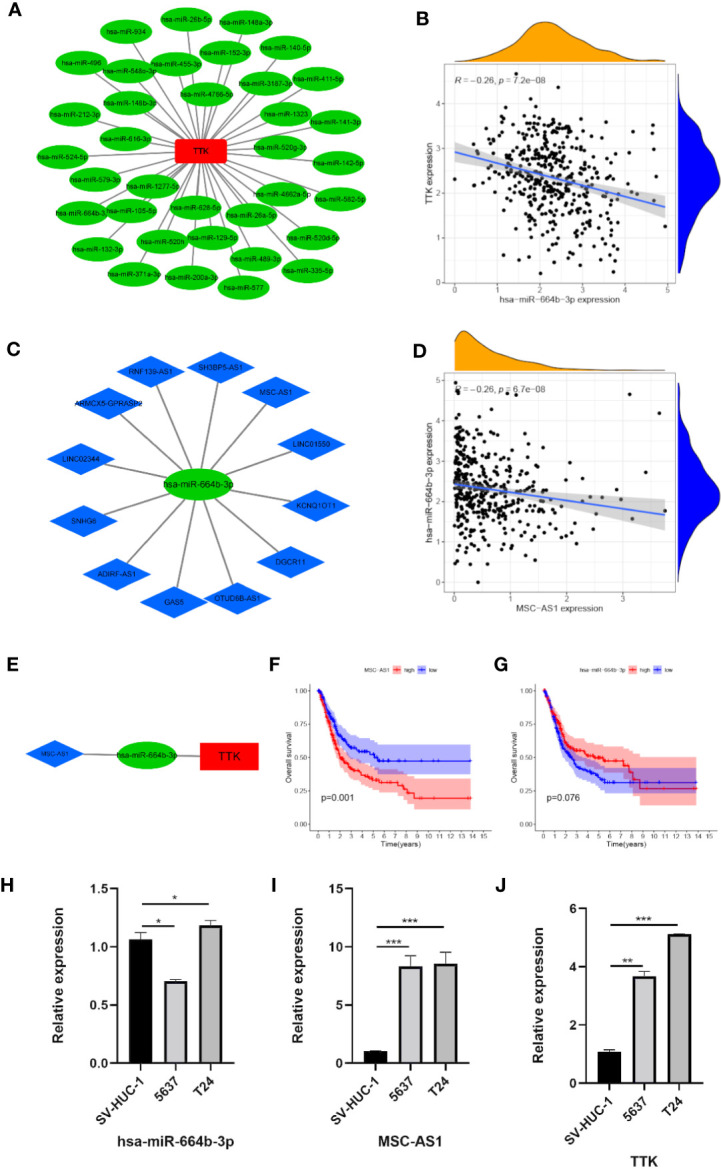
Construction of the lncRNA–miRNA–*TTK* network. **(A)** Results of the miRNA target prediction by starBase v3.0. **(B)** Correlation of *TTK* and hsa-miR-664b-3p. **(C)** Results of the lncRNA targets predicted by starBase v3.0. **(D)** Correlation of hsa-miR-664b-3p and MSC-AS1 expression. **(E)** The lncRNA–miRNA–*TTK* axis. **(F, G)** Prognostic value of MSC-AS1 **(F)** and hsa-miR-664B-3P **(G)** expressions in The Cancer Genome Atlas (TCGA) database. **(H–J)** Expressions of hsa-miR-664b-3p **(H)**, MSC-AS1 **(I)**, and TTK **(J)** in SV-HUC-1, 5637, and T24 cells. *p < 0.05; **p < 0.01; ***p < 0.001.

To elucidate the potential molecular mechanism by which *TTK* functions in BC, we constructed an mRNA–miRNA–lncRNA interaction network. According to starBase v3.0, we identified 36 miRNAs associated with *TTK* (**Figure 10A**). A negative Pearson’s correlation (Pearson’s *R* < −0.2, *p* < 0.05) was implemented to identify the potential miRNAs that could target *TTK* in TCGA, and we then identified hsa-miR-664b-3p (*R* = −0.26, *p* < 0.05; **Figure 10B**). Thereafter, we identified the upstream lncRNA MSC-AS1 of hsa-miR-664b-3p by the same method (*R* = −0.26, *p* < 0.05; **Figures 10C, D**). The lncRNA–miRNA–*TTK* axis is shown in **Figure 10E**. Through the survival analysis in TCGA, we found that the high expression of MSC-AS1 predicted poor OS of BC patients (*p* = 0.001; **Figure 10F**), while the low expression of hsa-miR-664b-3p seemed to predict poor OS; unfortunately, the *p*-value was >0.05 (*p* = 0.076; **Figure 10G**), so more samples must be studied. In addition, we found that, compared with that in the normal bladder epithelial cell SV-HUC-1, the expressions of *TTK* and MSC-AS1 were higher in the BC cell lines 5637 and T24, while those in SV-HUC-1 cells were higher than those in 5637 cells and lower than the expression level in T24 cells (*p* < 0.05; **Figures 10H–J**). The results also showed that there was a potential negative correlation between the expression levels of hsa-miR-664b-3p and those of its upstream and downstream genes. Thus, the lncRNA MSC-AS1/hsa-miR-664b-3p/*TTK* regulatory axis may play a vital role in the progression of BC, and this requires more data samples and further experiments.

## Discussion

Although the treatment of BC has improved, the prognosis of patients is generally poor due to the lack of accurate molecular targets. Therefore, better biomarkers are needed to specifically predict the prognosis and progression of BC. In this study, 40 genes with the same expression trend were identified in the TCGA and GSE13507 datasets by comprehensive bioinformatics analysis. According to the MCC scores of the CytoHubba plug-in in Cytoscape, the first 10 genes (*TROAP*, *CENPF*, *PRC1*, *AURKB*, *CCNB2*, *CDC20*, *TTK*, *CEP55*, *ASPM*, and *CDCA8*) related to BC were screened. Among these genes, the high expression of *TTK* was significantly correlated with the poor OS rate of patients with BC. We suggest that *TTK* may play a role in promoting the occurrence and development of BC.

*TTK* is a dual-specific protein kinase that phosphorylates serine/threonine and tyrosine (24). It is the core component of the spindle assembly checkpoint (SAC), and the SAC is a key monitoring mechanism that prevents the abnormal division of chromosomes by delaying the process of mitosis until all chromosomes are correctly attached to the spindle microtubules; this mechanism can ensure the accurate separation of chromosomes. Inactivation of the SAC will lead to the early withdrawal of the mitotic point, which will eventually lead to chromosome instability, aneuploid formation, and even cell death. The SAC can ensure the healthy growth and precise division of cells (25, 26). The abnormal expression of *TTK* inevitably affects the function of SAC (27). A high *TTK* expression can easily be found in several types of human malignant tumors (28–30). In addition, inhibition of *TTK* expression can suppress the proliferation of cancer cells and result in significant survival benefits (29, 31).

To explore the potential mechanism underlying the carcinogenic effects of *TTK*, we used its median expression in 406 TCGA-BC samples as the cutoff point, and samples with high and low expressions of *TTK* were analyzed by GSEA. The GSEA results showed that the samples with high *TTK* expression were enriched in signaling pathways closely related to tumorigenesis and development, such as the PI3K/AKT/mTOR pathway, which also suggested the potential mechanism by which *TTK* promotes the occurrence and development of BC. The GSEA of immune function suggested that *TTK* might exert a potential effect on the TME in BC. Then, according to the difference and correlation analyses between *TTK* and immune-infiltrating cells, we found that *TTK* expression was inversely proportional to the number of plasma cells, Tregs, and resting mast cells and directly proportional to the number of M1 macrophages and activated mast cells. Among these cells, high numbers of plasma cells and Tregs were associated with better OS in BC, while high numbers of activated mast cells could lead to poor prognosis (32, 33). Therefore, *TTK* may affect the TME by regulating the number of these immune cells, thereby affecting the prognosis of patients with BC. This conclusion requires further research and validation.

To verify the above results, we first found that *TTK* was relatively highly expressed in human BC tissues by IHC, and qPCR confirmed its relatively high expression in the human BC cell lines 5637 and T24 compared with the immortalized human bladder epithelial cell line SV-HUC-1. Then, we knocked down *TTK* expression in 5637 and T24 cells to assess cell proliferation, migration, and invasion, and we found that 5637 and T24 cells with low *TTK* expressions had decreased proliferation, migration, and invasion. Subsequently, we also built an mRNA–miRNA–lncRNA network and determined an MSC-AS1/hsa-miR-664b-3p/TTK regulatory axis. MSC-AS1 can promote the occurrence and development of many kinds of tumors (34–36), and hsa-miR-664b-3p also plays a role in the occurrence and development of tumors (37, 38). In our study, we also found that the differences in the expressions of MSC-AS1, hsa-miR-664b-3p, and *TTK* in SV-HUC-1 cells, 5637 cells, and T24 cells were also approximately consistent with the regulatory relationship of the MSC-AS1/hsa-miR-664b-3p/*TTK* regulatory axis. All these pieces of evidence suggest that the MSC-AS1/hsa-miR-664b-3p/TTK regulatory axis may play an important role in the progression of BC. Further research should be carried out to verify this result.

In summary, we provided a comprehensive bioinformatics analysis to identify potential predictive biomarkers between BC and normal tissues. In this study, TCGA and GEO databases confirmed that there was a significant correlation between the expression of *TTK* and OS in patients with BC. Our findings provide new insights into the role of potential biomarkers of BC and suggest that these findings may have important clinical significance. However, our article also has several limitations. Firstly, the expression and risk prediction ability of *TTK* have not been verified in a large number of clinical samples. Secondly, the molecular mechanism by which *TTK* functions in BC remains to be explored by experiments. Moreover, single-cell omics and spatial transcriptomics may be applied in future studies. The establishment of new models and algorithms allows information from various single-cell omics databases to be compatible and integrated (39, 40). Applying bioinformatics methods and mining these data can provide a new perspective for further exploring the mechanism of *TTK* in BC. In addition, these findings may not apply to every BC subtype, and we need to conduct experiments to explore this in the future.

## Data Availability Statement

The original contributions presented in the study are included in the article/**Supplementary Material**. Further inquiries can be directed to the corresponding authors.

## Ethics Statement

The studies involving human participants were reviewed and approved by the Medical Ethics Committee of Renmin Hospital of Wuhan University. The patients/participants provided written informed consent to participate in this study.

## Author Contributions

KC designed this study. KC, JX, and WY performed the data analysis, prepared the figures, and wrote the manuscript. YX revised the content. FC and TR were responsible for confirming the authenticity of the data. All authors read and approved the final manuscript.

## Funding

The current study was funded by the Algorithm and Application of Intelligent Medical Service Based on Health-Related Big Data (grant no. 2019AEA170).

## Conflict of Interest

The authors declare that the research was conducted in the absence of any commercial or financial relationships that could be construed as a potential conflict of interest.

## Publisher’s Note

All claims expressed in this article are solely those of the authors and do not necessarily represent those of their affiliated organizations, or those of the publisher, the editors and the reviewers. Any product that may be evaluated in this article, or claim that may be made by its manufacturer, is not guaranteed or endorsed by the publisher.
