# Supplementary material for: Identification and Validation of Hub Genes Associated with Bladder Cancer by Integrated Bioinformatics and Experimental Assays
Source: Front Oncol. 2021 Dec 20;11:782981. doi: 10.3389/fonc.2021.782981 (PMC8721040; doi:10.3389/fonc.2021.782981)
Supplement: Supplementary file 1 [file Table_1.docx]

Supplemental information

Table S1. Information of datasets used in this study.

| Variables | Number | | |
| --- | --- | --- | --- |
|  | TCGA-BC | GSE13507 | GSE32894 |
| **Total** | 408 | 165 | 308 |
| **Age** |  |  |  |
| <65 | 150 | 96 | 220 |
| ≥65 | 256 | 69 | 88 |
| **Gender** |  |  |  |
| Male | 299 | 135 | 80 |
| Female | 107 | 30 | 228 |
| **Grade** |  |  |  |
| High grade | 383 | 60 | - |
| Low grade | 20 | 105 | - |
| Unknown | 3 | - | 3 |
| Grade I | - | - | 48 |
| Grade II | - | - | 103 |
| Grade III | - | - | 154 |
| **Stage (TNM**) |  |  |  |
| Stage I | 2 | 103 | - |
| Stage II | 129 | 1 | - |
| Stage III | 140 | 0 | - |
| Stage IV | 133 | 1 | - |
| Unknown | 2 | 60 | - |
| **T** |  |  |  |
| T0 | 1 | - | - |
| Ta | - | 24 | 116 |
| T1 | 3 | 80 | 97 |
| T2 | 118 | 31 | 85 |
| T3 | 193 | 19 | 7 |
| T4 | 58 | 11 | 1 |
| Unknown | 33 | - | 2 |
| **N** |  |  |  |
| N0 | 236 | 149 | 49 |
| N1-3 | 128 | 15 | 22 |
| Unknown | 42 | 1 | 237 |
| **M** |  |  |  |
| M0 | 195 | 158 | - |
| M1 | 11 | 7 | - |
| Unknown | 200 | - | - |

Table S2. Primer sequence.

| Gene | Forward primer | Reverse primer |
| --- | --- | --- |
| TTK | 5’-AAACAGTGTTCCGCTAAGTGATG-3’ | 5’-AGGGCAATTTCCAGCATTTCTA-3’ |
| MSC-AS1 | 5’-GCAGACTTCAAATCATCCCAACA-3’ | 5’-CCACAATGCTGACATAGTCCTGAA-3’ |
| hsa-miR-664b-3p | 5’-ACACTCCAGCTGGGTTCATTTGCCTCCCAG-3’ | 5’-TGGTGTCGTGGAGTCG-3’ |
| GAPDH | 5’-GGAAGCTTGTCATCAATGGAAATC-3’ | 5’-TGATGACCCTTTTGGCTCCC-3’ |
| U6 | 5’-CTCGCTTCGGCAGCACA-3’ | 5’-AACGCTTCACGAATTTGCGT-3’ |

Table S3. The 40 overlapping genes.

| Gene |
| --- |
| APCDD1 |
| ASF1B |
| ASPM |
| AURKA |
| AURKB |
| CCNB2 |
| CDC20 |
| CDCA3 |
| CDCA5 |
| CDCA8 |
| CDT1 |
| CENPF |
| CEP55 |
| CKAP2L |
| DTL |
| E2F2 |
| FABP4 |
| GINS2 |
| IGFBP2 |
| IQGAP3 |
| KIF20A |
| KIF2C |
| NCAPG |
| NUSAP1 |
| PAQR4 |
| POLQ |
| PRC1 |
| RAD54L |
| RECQL4 |
| SCN11A |
| SPAG5 |
| TACC3 |
| TK1 |
| TOP2A |
| TPX2 |
| TRIP13 |
| TROAP |
| TTK |
| UBE2C |
| UHRF1 |
